# Effect of Tai Chi exercise on lower limb function and balance ability in patients with knee osteoarthritis: A protocol of a randomized controlled trial

**DOI:** 10.1097/MD.0000000000027647

**Published:** 2021-11-19

**Authors:** Haoyun Zheng, Dong Zhang, Yonggang Zhu, Qingfu Wang

**Affiliations:** a Beijing Hospital of Traditional Chinese Medicine, Beijing, China; b Dongfang Hospital Beijing University of Chinese Medicine, Beijing, China; c Beijing University of Chinese Medicine Third Affiliated Hospital, Beijing, China.

**Keywords:** knee osteoarthritis, lower limb function and balance ability, randomized controlled trial, Tai Chi

## Abstract

**Background:**

Knee osteoarthritis (KOA) is a chronic degenerative joint disease commonly occurring in middle-aged and elderly people. The main clinical manifestations are joint pain, limited activity, and decreased muscle strength resulting in decreased motor control ability. Exercise therapy is an effective method to enhance muscle strength of lower limbs, while China's traditional skill Tai Chi (TC) is a combination of activity and inertia, internal and external exercise therapy. In recent years, scholars at home and abroad have found that regular TC can effectively improve patients’ lower limb function and balance ability. The purpose of this study is to explore the effects of TC on lower limb function and balance ability in patients with KOA.

**Methods:**

This is a prospective randomized controlled clinical trial. One hundred forty-six cases of KOA patients will be randomly divided into experimental group and control group according to 1:1 ratio, 73 cases in each group, the control group: sodium hyaluronate; experimental group: TC added on the basis of the control group. Both groups will receive standard treatment for 5 weeks and will be followed up for 3 months. Observation indicators include: the western Ontario and McMaster universities osteoarthritis index; hospital for special surgery knee score; balance stability index, liver and kidney function, adverse reaction rate, etc. SPSS 23.0 software will be used for data analysis.

**Discussion:**

This study will evaluate the effects of TC on lower limb function and balance ability of patients with KOA. The results of this trial will provide a clinical basis for the selection of exercise therapy for patients with KOA.

## 1 Introduction

Knee osteoarthritis (KOA) is the most common degenerative joint disease involving cartilage and surrounding tissues, which is characterized by a series of variation such as changes in articular cartilage bone, osteophyte formation, and decreased joint muscle vitality.^[1]^ Clinical manifestations include joint pain, deformity, and reduced function,^[2]^ increasing the risk of fracture and fall.^[3]^ KOA is one of the common causes of mobility disorders and seriously affects the quality of life of patients.^[4,5]^ With the aggravation of population aging, KOA has become a public health problem that endangers global human health.^[6]^ According to statistics, more than 1/3 of the elderly suffer from KOA, and the disability rate is as high as 53%.^[7]^ As for the effective treatment of KOA, a large number of clinical and basic studies have been carried out at home and abroad, mainly including drug therapy, exercise therapy, and surgical treatment,^[8]^ all of which are aimed at relieving pain, delaying disease progression, protecting knee joint, and improving patients’ quality of life. As an important part of non-drug surgical treatment, exercise therapy has been recommended by several clinical guidelines as an effective method for the treatment of KOA.^[9–11]^

Tai Chi (TC), one of the traditional Chinese skills, is a defensive exercise with moderate intensity.^[12]^ It has become one of the most popular forms of exercise, which is rich in content and easy to learn, and is not restricted by space and time. It also emphasizes the integration of essence, qi and spirit combined with breathing to achieve the purpose of cultivating body and mind, preventing and treating diseases when exercising. Studies have found that TC exercise can increase muscle strength, improve balance and sensitivity.^[13]^ In recent years, studies on the effect of TC exercise on lower limb function of KOA patients have increased at home and abroad,^[14–16]^ but its clinical efficacy is still unclear.

Therefore, we intend to evaluate the effects of TC exercise on lower limb function and balance ability in patients with KOA in this randomized controlled trial.

## 2 Methods

### 2.1 Study design

**Figure 1:**
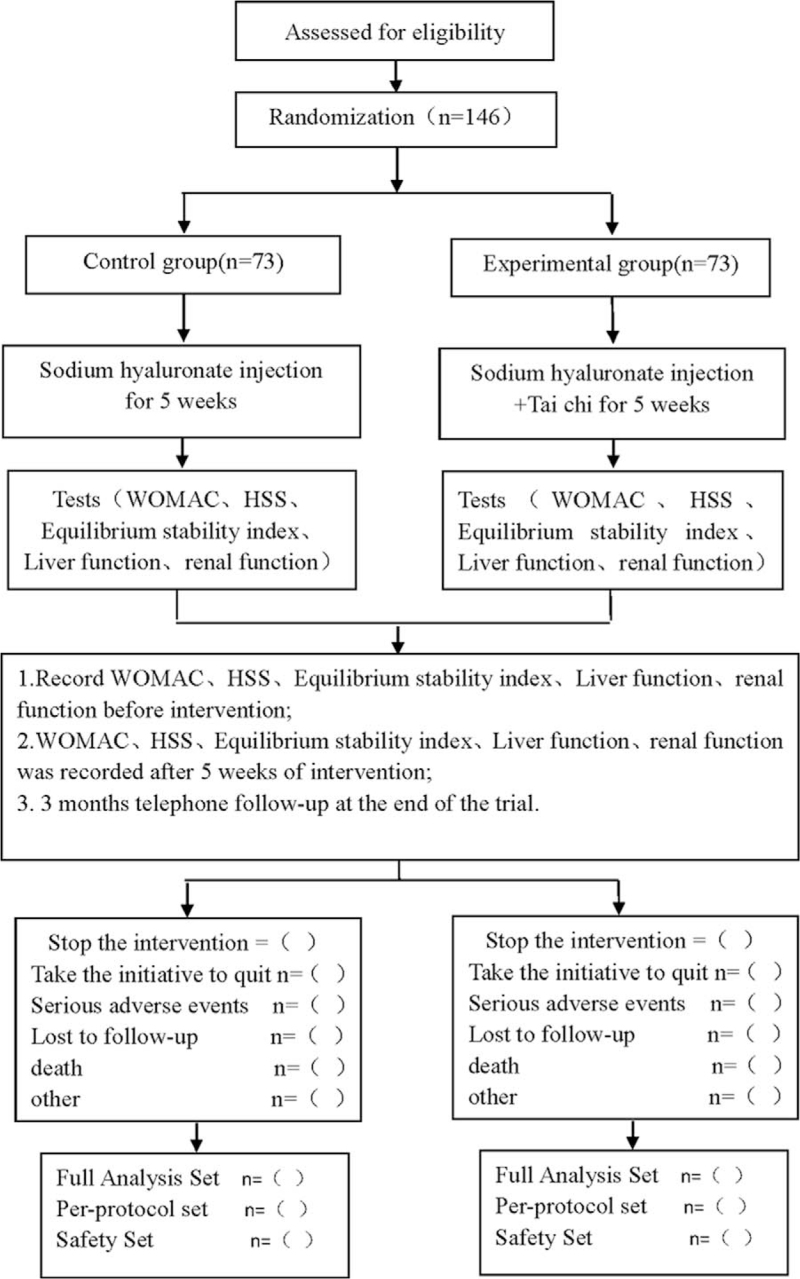
Flow diagram.

This is a prospective randomized controlled clinical trial studying the effects of TC on lower limb function and balance in patients with KOA. This protocol follows the latest Consolidated Standards of Reporting Trials (2017) (flowchart is shown in Fig. 1) and Standard Protocol Items: Recommendations for Interventional Trials 2013 statement.

### 2.2 Ethics and registration

This research scheme is in accordance with the Helsinki Declaration and approved by the Clinical Research Ethics Committee of our hospital. This study is registered in the open science framework with registration number of DOI 10.17605/OSF.IO/9WZ6Y. Before being randomly divided into groups, all patients are asked to sign a written informed consent form, and only patients who sign informed consent will be enrolled in the study.

### 2.3 Patients

Diagnostic criteria: revised according to the diagnostic criteria for KOA from the American College of Rheumatology^[17]^: ① Patients with knee pain for most of the month; ② Radiographs shows osteophyte formation along the joint margin; ③ Joint fluid examination is consistent with osteoarthritis; ④ Age: ≥40 years old; ⑤ Morning stiffness ≤30 minutes; ⑥ Patients who have bone frictions in joint movement. Those who meet the criteria of ① + ②, ① + ③ + ⑤ + ⑥, or ① + ④ + ⑤ + ⑥ can be diagnosed as KOA.

Inclusion criteria: ① Patients who meet the diagnostic criteria of KOA; ② Age: ≥40 and ≤80 years old; ③ Patients who have certain communication and understanding ability, and can express their own will correctly; ④ Patients who voluntarily accept corresponding treatment and can cooperate; ⑤ Patients who sign the informed consent.

Exclusion criteria: ① Patients who suffer from acute systemic illness; ② Patients who have severe impairment of limb function; ③ Patients who have relative or absolute contraindications of rehabilitation exercise such as blood pressure descent during exercise; ④ Patients combined with hematopoietic system abnormalities; ⑤ Patients combined with severe mental illness; ⑥ Patients who are allergic to sodium hyaluronate; ⑦ Patients who are unable to understand the study protocol or unwilling after explanation.

Elimination criteria: ① Patients with serious adverse events or serious complications, which is not suitable for the next step; ② Patients with poor compliance, affecting the judgment of effectiveness and safety; ③ Patients whose disease progresses during the treatment and need to change treatment plan; ④ Patients who ask to withdraw from the study for any reason.

### 2.4 Sample size

Since the main efficacy index of this study is the total effective rate, the sample size will be estimated according to the total effective rate of pre-experiment. According to the preliminary clinical pre-experiment, the total effective rate of TC combined with sodium hyaluronate was 91.3%, and that of sodium hyaluronate was 68.57%. PASS15.0 will be used for sample size estimation, and an optimal design will be adopted. α = 0.05, β = 0.2, test efficiency = 0.8, the number of experimental group: the number of control group = 1:1, threshold = –0.4. According to the software calculation, the total sample size of the 2 groups will be 130 cases, and 146 cases will be finally included considering the clinical shedding rate of about 10%. The included patients will be numbered according to the order of treatment and divided into the treatment group and the control group by completely random method, with 73 cases in each group.

### 2.5 Interventions

The study will select patients who meet the study criteria by recruiting them at the hospital. A randomized controlled study will be conducted, in which sodium hyaluronate will be used in the control group and the experimental group will include TC exercise on the basis of the control group. Patients in both groups will receive the same routine care, avoid alcohol, tobacco and irritating food, avoid strenuous exercise, and adverse reactions in patients during the test need to be concerned. If necessary, the attending doctor may adjust the treatment according to the patient's condition, and all interventions will be recorded in detail for final outcome analysis. Efficacy assessors are not aware of the study plan, and data statisticians are not involved in study design and implementation. The health of each patient will be assessed before and after the treatment, including observational indicators, and all patients will be followed up by telephone. The follow-up visit includes cardiovascular events and re-hospitalization.

(1) Control group: patients will be placed in the sitting or supine position, and the knee joint will be kept bent 70° to 90°, sterilized strictly, with sterile towel laid and aseptic operation performed. The lower lateral or inner side of the patella will be used as the conventional puncture point, and the knee joint cavity will be punctured through a 5 mL syringe. If there is joint effusion, it will be drained first, and then 20 mg sodium hyaluronate will be injected (Shandong Bausch Fruida Pharmaceutical Co., LTD., specification 2 mL:20 mg, CFDA approval number: H10960136); if not, 20 mg sodium hyaluronate can be directly injected into the joint cavity. After injection, help the patient to move the knee joint so that sodium hyaluronate can be evenly coated on the internal surface of the joint. One time/week, 5 times as a course of treatment. Health education should be carried out for patients to avoid acute jumping, walking up and down stairs, climbing mountains and other activities that damage joints, and remind them to pay attention to the warmth of joints as well. Pay attention to communicate with patients, be aware of their psychological and emotional changes, avoid or relieve anxiety, depression, and other adverse emotions of the elderly patients.
(2) Experimental group: The experimental group will be given 24 simplified TC on the basis of conventional treatment. The subjects will be taught and practiced by professional TC teachers to ensure that the practice time and movement rhythm are basically consistent. The complete set of TC consists of 3 parts: preparation, training, and ending activities. It lasts for about 20 minutes per time, twice a day, once in the morning and once in the afternoon, and lasts for 5 weeks in total.

### 2.6 Outcomes

#### 2.6.1 Observation indicator

① Use Western Ontario and McMaster Universities Osteoarthritis Index^[18]^ and choose V3.1 Mandarin version. A total of 24 items will be evaluated in terms of pain, stiffness, and joint function. The value of each item will be recorded by visual analogue scale.^[19]^ The higher the score, the more serious the disease. ② Hospital for special surgery knee score^[20]^ will be used to evaluate the knee function of patients, and the total score of hospital for special surgery knee score is 0 to 100 points. The higher the score, the better the knee function of patients. ③ Balance index: Italian Tecnobody dynamic balance tester will be used to record the balance data of patients during dynamic trajectory measurement in B position (standing position, 50% load on 1 leg) before and after training. Items include anterior and posterior axis stability index, left and right axis stability index, A2 to A6 axis stability index, A4 to A8 axis stability index, and circular axis stability index before and 5 weeks after the treatment. ④ Hepatorenal function.

#### 2.6.2 Efficacy indicator

Main efficacy indicators: total effective rate: according to *Guiding Principles for Clinical Research of New Chinese Medicine*,^[21]^ total effective rate = (clinical control + significantly effective number + effective number)/total number of people × 100%. ① Clinical control: pain and other symptoms disappear, joint activity is normal, integral reduction ≥95%; ② Significantly effective: pain and other symptoms disappear, joint activity is not limited, 70% ≤ integral reduction < 95%; ③ Effective: pain and other symptoms basically disappear, joint activity is slightly limited, 30% ≤  integral reduction < 70%; ④ Ineffective: no significant improvement in symptoms such as pain and joint activity, integral reduction < 30%.

Secondary efficacy indicators: changes in osteoarthritis index score, knee joint score, and balance index

#### 2.6.3 Adverse event rate

Including the number of patients who experience any uncomfortable symptoms during treatment (such as dizziness, nausea, etc).

### 2.7 Sample collection

Data will be collected according to the evaluation criteria before and 5 weeks after the treatment, and telephone follow-up will be conducted for 3 months after the treatment. If follow-up information cannot be collected, the reasons for loss of follow-up shall be recorded in detail. Access to the database is restricted to the researchers in this research group.

### 2.8 Study quality control

Throughout the trial, each participant will be monitored for safety. All adverse event occurrences will be referred to the office of the Clinical Research Ethics Committee, which will review the events and determine causal relationship. We will set Data and Safety Monitoring Board. Members of Data and Safety Monitoring Board include physicians, clinical pharmacists, trial method experts, statistical experts, and members of the ethics committee, who will conduct risk assessment and safety analysis procedures according to termination conditions.

### 2.9 Statistical analysis plan

Use Excel to build database. Full analysis set: including all subjects randomly enrolled, treated and visited at least once, and use full analysis set for intent-to-treat analysis. Per-protocol set: including all subjects who comply with the trial protocol, have no missing baseline variables, and have measurable major variables. Safety set: including subjects randomly enrolled, treated at least once and being safe after at least 1 treatment.

SPSS25.0 statistical analysis software will be used for data analysis in this study. If measurement data meet normal distribution, independent-sample *t* test will be used between groups and paired-sample *t* test will be used within groups; if not, non-parametric test will be used and the results will be expressed in quartiles; Chi-square test will be used for counting data. The incidence of adverse events is compared by chi-square test. When *P* *<* *.05*, the difference is statistically significant.

## 3 Discussion

Clinically, non-steroidal anti-inflammatory drugs, glucocorticoids, intra-articular injection of sodium hyaluronate, and other related drugs are usually used for the treatment of KOA patients, which can relieve pain in a short term,^[22]^ but the efficacy of these drugs is not stable. In addition, it has different degrees of gastrointestinal reactions, liver and kidney damage, toxic and side effects on chondrocytes, and other adverse reaction.^[23,24]^ Sodium hyaluronate is a physiologically active substance widely found in human body with high viscosity. Under the condition of abnormal stress of articular cartilage, it can reduce vibration and play a role in lubrication and joint protection.^[25]^ Injection of sodium hyaluronate can significantly improve the inflammatory response of synovial fluid tissue and delay the progression of KOA.^[26]^ Clinical studies have confirmed that joint dysfunction and reduced balance ability can be improved through exercise training.^[27]^

TC is regarded as a kind of exercise that alternates muscle contraction training with total body relaxation. Studies have shown that long-term and regular TC exercise can effectively improve static balance and coordination ability,^[28,29]^ increase the strength of periarticular muscle groups through resistance exercises,^[30]^ relieve joint pain,^[31]^ relieve joint stiffness,^[32]^ and promote the recovery of joint function, which is superior to non-drug surgical treatments such as hydrotherapy.^[33]^ Some scholars believe that TC can activate neuroendocrine and autonomic nervous functions, secrete neurochemicals, cause behavioral responses in analgesic pathways, and then regulate inflammatory responses of the immune system.^[34,35]^ In the process of training, the trainees always keep the posture of slightly bent knees and horse-riding step. With the changes of movement, the center of body rotates constantly, which is conducive to self-repair and stability mechanism, release endogenous neurohormones, and achieve the purpose of maintaining balance.^[36]^ At present, no serious adverse events related to TC have been reported.^[37,38]^ As a kind of KOA exercise therapy, TC is widely recognized as a kind of exercise therapy for KOA patients.

The purpose of this randomized controlled trial is to examine the effects of TC on lower extremity function and relaxation ability in patients with KOA. Since there is no standard large-sample clinical study to evaluate the effects of TC on lower extremity function and relaxation ability in patients with KOA, we intend to evaluate its efficacy in a prospective randomized controlled study.

There are also some limitations of this study: the short follow-up period does not allow us to understand the impact of long-term outcomes, so we may extend the follow-up period if necessary.

## Author contributions

**Data collection:** Haoyun Zheng and Dong Zhang

**Investigation:** Haoyun Zheng and Yonggang Zhu

**Resources:** Dong Zhang and Yonggang Zhu

**Software operating:** Haoyun Zheng and Dong Zhang

**Writing – original draft:** Haoyun Zheng and Qingfu Wang

**Writing – review and editing:** Dong Zhang and Qingfu Wang

**Funding support:** Qingfu Wang

**Supervision:** Yonggang Zhu

**Data curation:** Haoyun Zheng.

**Funding acquisition:** Qingfu Wang.

**Investigation:** Yonggang Zhu.

**Resources:** Dong Zhang.
